# Supplementary material for: Sugarcane Streak Mosaic Virus P1 Attenuates Plant Antiviral Immunity and Enhances Potato Virus X Infection in Nicotiana benthamiana
Source: Cells. 2022 Sep 14;11(18):2870. doi: 10.3390/cells11182870 (PMC9497147; doi:10.3390/cells11182870)
Supplement: Supplementary file 1 [file cells-11-02870-s001.zip › cells-1854153-supplementary materials/cells-1854153-Supplementary Figures.pdf]

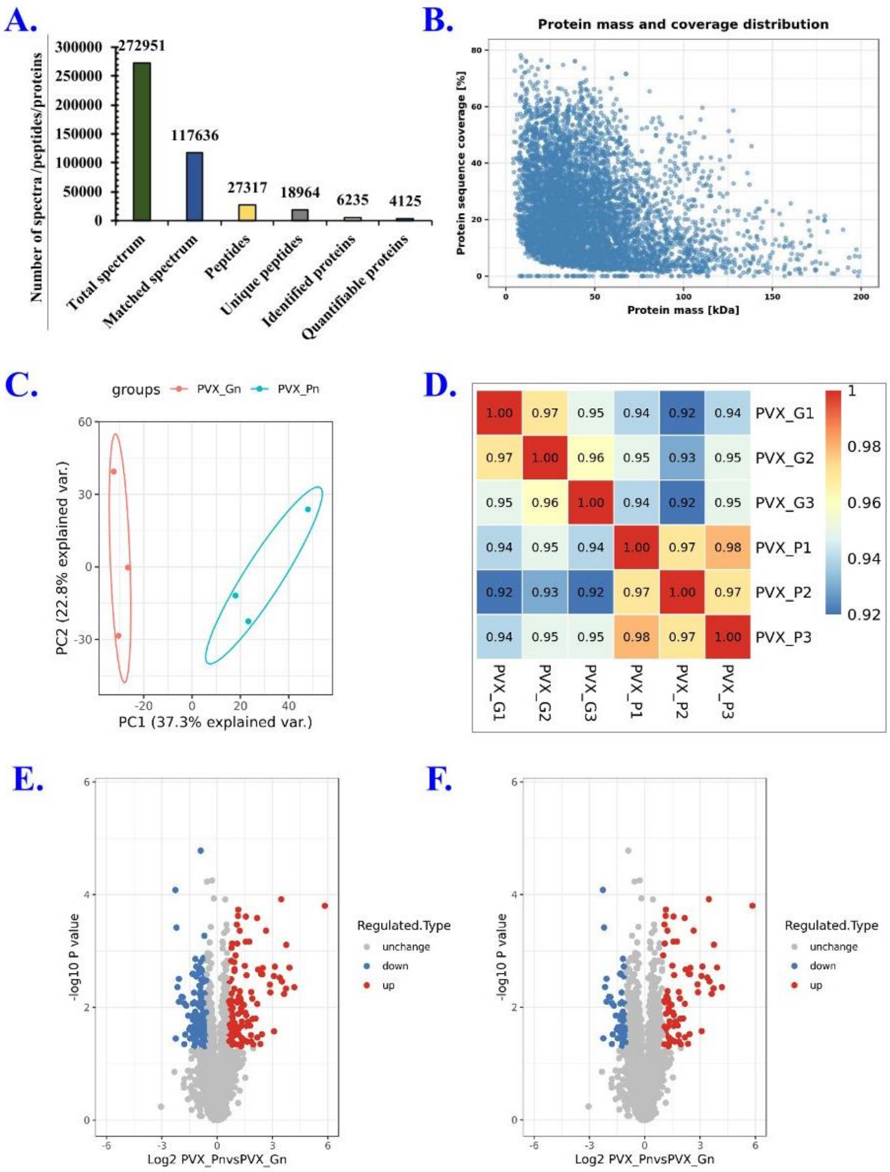

Figure S1. Results of 4D-proteomics, quality control, and differentialy abundant proteins (DAPs). (**A**). Identified spectra after reversed-phase HPLC and LC-MS/MS with 27317 total peptides. Proteins were identified by searching and analyzing *N. benthamiana* protein and UniProt databases. The Y-axis shows the number of spectra, peptides, and proteins. (**B**). Relationship and of protein mass and coverage. The X-axis represents the protein mass, and the Y-axis shows the sequences coverage of the target protein. (**C**). Quality control of 4D-proteomics by principal component analysis. The red dots circled refer to the PVX-GFP group, and the cyan dots represent the PVX-P1^{SCSMV} group. (**D**). Quality control of 4D-proteomics by Pearson's correlation coefficient. (**E**). Numbers of DAPs among the PVX-GFP and PVX-P1^{SCSMV} groups equal to or greater than 1.5 -fold. Red spot: upregulated proteins. Blue spot: downregulated proteins. Log2 PVX_Pn vs PVX_Pn represents the ratio between PVX-GFP and PVX-P1^{SCSMV} groups taking the natural logarithm. The Y-axis indicates the magnitude of differences in protein levels. (**F**). Number of DAPs among the PVX-GFP and PVX-P1^{SCSMV} groups equal to or greater than 2-fold.

A.

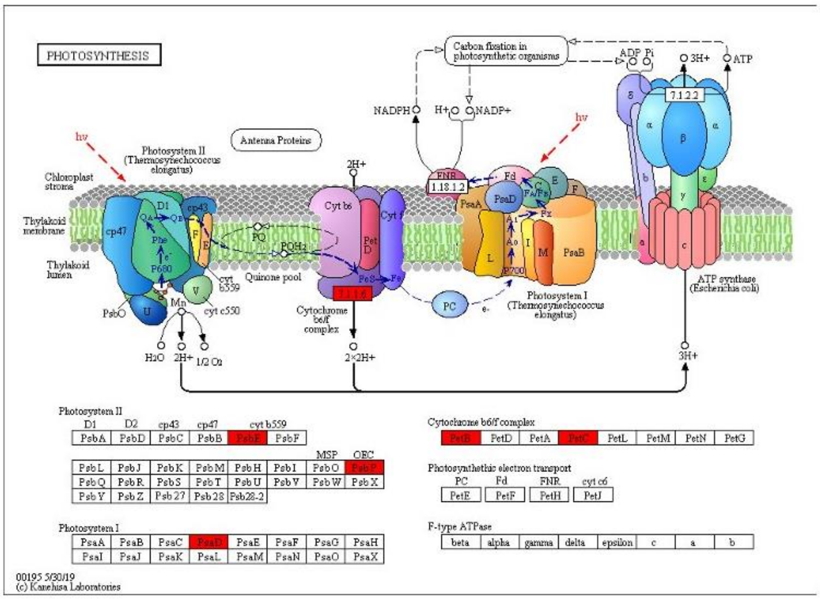

B.

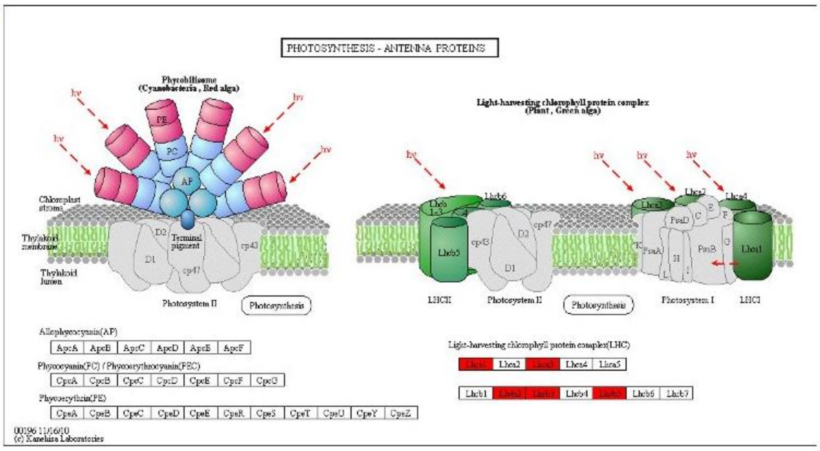

Figure S2. Expression profile of photosynthesis-related proteins by 4D-proteomics. (**A**). Photosynthesis map illustrating directly related proteins, including photosystem I, photosystem II, cytochrome b6/f complex, photosynthetic electron transport, and F-type ATPase. (**B**). Photosynthesis map illustrating the photosynthesis related antenna proteins. All the red marked proteins were significantly upregulated by P1^{SCSMV} overexpression.
