# Supplementary material for: Sugarcane Streak Mosaic Virus P1 Attenuates Plant Antiviral Immunity and Enhances Potato Virus X Infection in Nicotiana benthamiana
Source: Cells. 2022 Sep 14;11(18):2870. doi: 10.3390/cells11182870 (PMC9497147; doi:10.3390/cells11182870)
Supplement: Supplementary file 1 [file cells-11-02870-s001.zip › cells-1854153-supplementary materials/Table S1.pdf]

Supplementary Table S1 List of the specific pairs of primer corresponding to selected differentially abundant proteins for qRT-PCR in *Nicotiana benthamiana*

Protein Name[a]	Protein ID[b]	Transcription ID[c]	Primer sequences (5'-3')	Length of PCR products (bp)	Tm value (°C) [d]
5-epi-aristolochene synthase-like (EAS)	NbS00004447g0101.1	Niben101Scf03993g05005.1	GGAAGGAAGACGGCTGATACG<br>CGCAGTTTGAGGTTTGGTTGTA	124	60
Endochitinase A	NbS00023821g0011.1	Niben101Scf07491g00003.1	ATGGGGTTATTGCTGGCTTAG<br>CCACAAGGTCCGTAGTTATAGTTG	144	58
Aspartic proteinase (ASP)	NbS00002190g0213.1	Niben101Scf04084g00005.1	CCATTGCCTGCTTTGTACTTC<br>GCCCTTCATCACATGCTTTC	143	57
Xyloglucan endotransglucosylase (XTH1)	NbS00056252g0003.1	Niben101Scf00369g05011.1	GCCCCATTTACTGCCTCCTA<br>GCCTTTTGATCCCACCATCT	113	58
PR-1 like (PR-1)	NbS00061216g0001.1	Niben101Scf04053g02006.1	ATGGGACAATAGGGTAGCGG<br>GGCGTTGAGTTGAGGGAAGG	127	59
Catalase (CAT)	NbS00013764g0007.1	Niben101Scf14996g00009.1	CTTCGATGATGTTTGTCTCCCG<br>GCTTTCCCCGCCTTGTTGAT	90	62
Glutathione S-transferase U9-like (GST)	NbS00003017g0007.1	Niben101Scf10254g01002.1	AAGGTCCCTGTGCTTGTCCA<br>TAATTAGCCCAGAAACGAACTATG	146	58
L-ascorbate oxidase	NbS00052235g0008.1	Niben101Scf03026g01009.1	TTGTGAACCCAATTATGAAGAACA<br>GACTCAATATGGCAATGGAAGG	112	59
Lycopene beta cyclase	NbS00023329g0001.1	Niben101Scf06266g00002.1	GGTTCATCCCTCAACTGGTTAT<br>TCTATGGGCCACAAAATCTTTC	150	58
Cell division protein FtsZ	NbS00028082g0012.1	Niben101Scf07163g00020.1	TATTCGGTGCTGTTGTGGATG<br>TGATACCGTTCTTTCCGTAGTTC	158	58
PsbP domain-containing protein 5	NbS00055576g0008.1	Niben101Scf00777g04013.1	GGTGAGCCCTATTGGTACTATGA<br>GGAAGAAGTCGGAAAGATGCC	156	58
Dynamin-like protein ARC5 isoform	NbS00032594g0011.1	Niben101Scf08364g00011.1	ATGTATTTCTGAGGCGTGTAGCA<br>CCGATTCTTATTGTGGAGGGAC	137	59
Elongation factor 1α (EF1α)	NbS00019623g0001.1	Niben101Scf08618g01001.1	TAAGATGATGCCGACCAAGC<br>TGTCCCTCACAGCAAAACGA	110	58

[a] General proteins names

[b] The accession number of *N. benthamiana* proteins database (https://solgenomics.net/)

[c] The accession number of *N. benthamiana* transcripts database (https://solgenomics.net)

[d] The Tm value of qRT-PCR used in the study
