# Supplementary material for: Sugarcane Streak Mosaic Virus P1 Attenuates Plant Antiviral Immunity and Enhances Potato Virus X Infection in Nicotiana benthamiana
Source: Cells. 2022 Sep 14;11(18):2870. doi: 10.3390/cells11182870 (PMC9497147; doi:10.3390/cells11182870)
Supplement: Supplementary file 1 [file cells-11-02870-s001.zip › cells-1854153-supplementary materials/Table S2.pdf]

Supplementary Table S2-List of the specific pairs of primers for selected candidate genes that the corresponding protein directly interacted with the PVX encoded proteins

Protein Name[a]	Transcription ID[b]	Primer Sequences (5'-3')	Length of PCR Products (bp)	Tm value (ºC)[c]
NbeEF1A	Niben101Scf07423g04011.1	AAAATCCCCTTTGTCCCC	94	56.0
		TTTCGATCATGTTATCACCCTC		56.8
NbeEF1Bβ-1	Niben101Scf19454g00004.1	CTAGCAAAAGCCCCATCA	90	54.2
		TCCCCAGATACACCACAGAT		54.5
NbeEF1Bβ-2	Niben101Scf00337g03005.1	GTGGCTATACTGAATAACAACAATC	89	55.4
		GCATAACAGATACCCTTGACGA		57.2
NbTIPs	Niben101Scf02016g00005.1	AGGAGGTCCTAAAACTGCTCG	92	58.1
		AAACCCCTGTGACATCCATAA		56.9
NbFD1	Niben101Scf22688g00018.1	AAACGATTAAAACCCCGAACT	88	57.8
		TACGCAACAACCGTGACCT		56.9
NbREM1	Niben101Scf15568g00037.1	GTGACGAGCTGAAGTTGATGTA	94	55.4
		GTTTTGGCTTTTGTAGTGGC		55.3
NbREM2	Niben101Scf08587g00002.1	TCTACCAGCAAGTTGATTCTCC	91	56.4
		TCCATCTCCTCCTTTACCGT		56.1
NbREM3	Niben101Scf04804g01002.1	TGCCTCCTCCTGAACAAGTC	86	57.2
		CAGCAGGTTTATCTTCTACGAC		54.1
NbREM4	Niben101Scf02910g01040.1	GCCTTAGTCCCATCTGTCAAC	91	56.2
		TCTCAGTCTCCACTTTCGTAGC		56.5
NbPlastocyanin	Niben101Scf00539g07027.1	CTCTGCTGCTGTTGCTATCC	93	55.9
		CTTTCAAAGAAGCCTTCACC		54.4
NbActin-1	Niben101Scf04140g01010.1	TGAAGATCAAGGTGGTGGC	83	56.0
		TGAAGATCAAGGTGGTGGC		57.8

NbActin-2	Niben101Scf00096g04015.1	AGGCTACTCTTTCACTACCACG	100	56.0
		CTTCTCCTTCACATCCCTAACA		56.3
NbActin-3	Niben101Scf03410g03002.1	TTGCTATTCAGGCTGTGCTC	98	56.4
		TCATCAGGCTATCGGTAAGG		55.3
NbPCIP1-1	Niben101Scf03143g06001.1	GTGGCTATGGAAGTGGAATG	94	55.2
		TCTGGCTGTACTGACTAGGGTG		57.1
NbPCIP1-2	Niben101Scf02720g00003.1	GCCAGTCCACTCACAATCAC	98	55.4
		CTGCCATAGCCAGCATCAC		57.0
NbPCIP1-3	Niben101Scf01317g02003.1	TGATGATGGGTCGTGGTAG	100	53.9
		GCTTGAAAAGTTGGTGGAGT		54.3
NbMPB2Cb-1	Niben101Scf00337g03002.1	ATCGTCCCTCTTGTTCAGTC	98	53.6
		TTGGTCCCTATGCTTCTTTG		55.4
NbMPB2Cb-2	Niben101Scf01531g03011.1	TCGTCCCTCTTGTTCAGTCC	98	57.1
		TTGGTCCCTATGCTTCTTTG		55.4
NbCPIP1	Niben101Scf02043g02002.1	GGGAACTATGAACCTGGAGC	85	56.1
		CCATCCCTCTTGAAGACAGC		56.9
NbCPIP2	Niben101Scf06958g01003.1	GGTGGAGAATAAACTGTCGTGT	96	56.0
		TGTAGGCTTGCCAGAGTCAT		55.5
NbCPIP3	Niben101Scf02806g00030.1	GTTCCCATCAAGGCTTAGTTC	94	55.8
		GTCAACAGTCCTGCCCAAC		55.4
NbCPIP4	Niben101Scf07875g00001.1	CCGTCAAGGCTTAGTTCAGA	89	55.5
		CATTGCACTTCCACAATCAGTT		57.7

[a] General proteins names

[b] The accession number of *N. benthamiana* transcripts database (https://solgenomics.net)

[c] The Tm value of qRT-PCR used in the study
