# Supplementary material for: Sugarcane Streak Mosaic Virus P1 Attenuates Plant Antiviral Immunity and Enhances Potato Virus X Infection in Nicotiana benthamiana
Source: Cells. 2022 Sep 14;11(18):2870. doi: 10.3390/cells11182870 (PMC9497147; doi:10.3390/cells11182870)
Supplement: Supplementary file 1 [file cells-11-02870-s001.zip › cells-1854153-supplementary materials/Table S4.pdf]

Supplementary Table S4-Differently abundant proteins in P1^{SCSMV} heterogeneous expressed *Nicotiana benthamiana* leaf by 4D-lable free proteomics.

Phytozome/ NCBI Accession[a]	Description[b]	Sequences coverage (%)[c]	Anova (p)[d]	Fold change[e]
**UP-accumulated proteins (70)**				
NbS00004447g0101.1	5-epi-aristolochene synthase-like isoform X1	40.3	0.00016	57.252
NbS00061216g0001.1	Basic form of pathogenesis-related protein	57.0	0.00440	18.169
NbS00023821g0011.1	Endochitinase A	51.9	0.00198	15.126
NbS00010282g0017.1	Beta-fructofuranosidase	19.6	0.00078	13.421
NbS00003709g0007.1	Glucan endo-1,3-beta-glucosidase	22.5	0.00464	13.288
NbS00034922g0015.1	Glucanase regulator	47.5	0.00580	12.361
NbS00045440g0005.1	Pathogenesis-related protein R	41.6	0.00348	11.448
NbS00003851g0014.1	Peroxidase	31.5	0.00012	11.121
NbS00005426g0113.1	NtPRp27	37.7	0.00298	10.538
NbS00018563g0001.1	Epoxide hydrolase	40.4	0.00190	8.726
NbS00000307g0001.1	Unnamed protein product	12.0	0.02662	8.507
NbS00035082g0005.1	Oxoglutarate/iron-dependent dioxygenase	36.4	0.00278	8.436
NbS00024994g0004.1	CYP92A2v4	17.4	0.00544	7.403
NbS00010102g0006.1	Secologanin synthase	19.9	0.00394	7.277
NbS00058225g0005.1	Peroxidase	26.9	0.00044	6.282
NbS00010545g0005.1	Conserved hypothetical protein	49.2	0.00200	5.549
NbS00013764g0007.1	Catalase isozyme 1	53.0	0.00258	5.494
NbS00010447g0101.1	Uncharacterized protein	32.4	0.02996	5.384
NbS00038999g0004.1	Pleiotropic drug resistance protein 1	7.9	0.00256	5.224
NbS00026613g0001.1	Osmotin	35.5	0.04462	5.074
NbS00013812g0011.1	Aminotransferase	16.2	0.00214	4.639
NbS00008099g0223.1	Glutathione S-transferase	27.8	0.03430	4.524
NbS00019212g0004.1	UDP-glycosyltransferase 74F2 [Vitis vinifera]	26.5	0.00870	4.520
NbS00010850g0119.1	Shikimate O-hydroxycinnamoyltransferase-like	24.3	0.00026	4.489
NbS00024411g0009.1	Acidic endochitinase Q	56.1	0.01584	4.236
NbS00053630g0004.1	Chloramphenicol acetyltransferase-like	13.6	0.04568	3.878
NbS00013552g0018.1	Phenazine biosynthesis-like domain-containing protein	43.6	0.01606	3.785
NbS00003017g0007.1	Glutathione S-transferase 12	26.5	0.00698	3.754
NbS00018998g0013.1	Bacterial-induced peroxidase precursor	21.0	0.04060	3.620
NbS00034398g0005.1	T-complex protein 1 subunit beta	15.1	0.00536	3.594
NbS00010129g0001.1	Glucan endo-1,3-beta-glucosidase	55.3	0.03116	3.563
NbS00010311g0003.1	Hydroxyacylglutathione hydrolase 3	32.0	0.02120	3.451
NbS00053961g0017.1	Lipoxygenase	19.6	0.00068	3.357
NbS00019371g0003.1	Peroxidase	13.6	0.01802	3.193
NbS00010078g0005.1	Chitinase	40.4	0.04066	3.089
NbS00051044g0009.1	Short-chain dehydrogenase	26.1	0.00270	3.017
NbS00022757g0014.1	Methyltransferase	51.3	0.02144	2.991
NbS00015135g0014.1	Hydroxymethylglutaryl-CoA synthase	34.8	0.01380	2.979
NbS00037921g0004.1	Argininosuccinate lyase	32.5	0.00024	2.947
NbS00016456g0007.1	Dehydrogenase/reductase SDR family member	26.1	0.00068	2.934
NbS00036561g0009.1	Proteinase inhibitor isoform	65.4	0.01252	2.891
NbS00052235g0008.1	Ascorbate oxidase precursor	17.9	0.00184	2.824

NbS00010099g0019.1	Agmatine deiminase, putative	22.5	0.00908	2.734
NP_054530.1	Cytochrome b6	15.3	0.03762	2.707
NbS00026556g0002.1	Hypothetical protein: hydrolase activity	21.5	0.03678	2.621
NbS00005033g0013.1	ClpP/crotonase-like domain	15.2	0.01772	2.531
NbS00000833g0023.1	Photosystem I P700 apoprotein A1	14.6	0.04198	2.529
NbS00008548g0006.1	Alternative oxidase [Nicotiana tabacum]	18.4	0.03435	2.462
NbS00004500g0004.1	Multidrug resistance protein ABC transporter family	18.4	0.01190	2.457
NbS00037999g0007.1	Phenylalanine ammonia-lyase	18.6	0.02158	2.457
NbS00017284g0011.1	NADP-dependent malic enzyme	16.2	0.04904	2.452
NbS00043723g0002.1	CYP81B2v2	31.9	0.01044	2.450
NbS00009732g0022.1	Monodehydroascorbate reductase	53.0	0.00816	2.438
NbS00011370g0002.1	Putative endo-1,3(4)-beta-glucanase 2	7.5	0.00714	2.414
NbS00023446g0007.1	Aldehyde/histidinol dehydrogenase	18.6	0.00518	2.352
NbS00019903g0005.1	RecName: Full=Pleiotropic drug resistance protein 1	10.2	0.00044	2.341
NbS00018742g0017.1	Enoyl-CoA hydratase	19.4	0.00640	2.272
NP_054517.1	Photosystem II protein V	48.2	0.02410	2.244
NbS00018522g0009.1	Upstream activation factor subunit spp27	23.6	0.03010	2.230
NbS00000692g0011.1	Cinnamyl alcohol dehydrogenase-like protein	33.0	0.01306	2.221
NbS00031166g0001.1	Probable carboxylesterase 17	50.6	0.00018	2.214
NbS00010140g0005.1	Hypersensitive-induced response protein	24.4	0.00024	2.185
NbS00001174g0003.1	Alanine aminotransferase	48.7	0.00816	2.122
NbS00038880g0004.1	Fumarase	29.7	0.00074	2.122
NbS00007839g0020.1	TOM1-like protein 2	22.6	0.02214	2.113
NbS00003479g0020.1	Catechol O-methyltransferase	55.9	0.00034	2.101
NbS00005741g0013.1	Formate--tetrahydrofolate ligase	30.1	0.04572	2.098
NbS00023745g0003.1	Glutamate decarboxylase isozyme 3	23.7	0.00774	2.072
NbS00006179g0007.1	3-ketoacyl CoA thiolase 1	40.0	0.00120	2.001
**Down-accumulated proteins (53)**				
NbS00013715g0011.1	Riboflavin biosynthesis protein ribBA	16.8	0.02212	0.495
NbS00014863g0008.1	PREDICTED: Tetratricopeptide-like helical domain	20.6	0.01572	0.495
NbS00058604g0005.1	PREDICTED: coatomer subunit β'-2-like	13.4	0.04184	0.485
NbS00028221g0013.1	Pale cress protein	18.5	0.00878	0.477
NbS00014416g0022.1	AMPK1_CBM domain-containing protein	14.5	0.02520	0.476
NbS00039424g0014.1	Protein HOTHEAD-like isoform X2	18.0	0.02575	0.471
NbS00057396g0007.1	Conserved hypothetical protein	18.1	0.02298	0.468
NbS00050387g0001.1	PREDICTED: 2-keto-3-deoxy-L-rhamnonate aldolase-like	12.8	0.01356	0.465
NbS00005939g0108.1	Pyrophosphate-energized vacuolar membrane proton pump-like	13.2	0.03286	0.462
NbS00056567g0001.1	PREDICTED: Protein BONZAI 1	14.9	0.01358	0.462
NbS00038786g0002.1	PREDICTED: pentatricopeptide repeat-containing protein	13.5	0.03030	0.459
NbS00012077g0011.1	Pectin acetylesterase	39.6	0.00190	0.456
NbS00057798g0003.1	GDSL lipase-like chlorogenate-dependent caffeoyltransferase precursor	19.7	0.00282	0.456
NbS00006643g0012.1	White-brown-complex ABC transporter family	8.2	0.02502	0.453
NbS00002134g0107.1	Tetrapyrrole-binding protein	12.7	0.03138	0.451
NbS00021029g0013.1	Cycloartenol synthase	16.5	0.04124	0.449
NbS00037540g0010.1	GcpE	31.5	0.02962	0.448
NbS00020149g0020.1	Translation factor GUF1 homolog	8.9	0.01726	0.444
NbS00039842g0002.1	P-glycoprotein	5.5	0.00138	0.441
NbC24809697g0001.1	Hypothetical protein MA29G21.11 [Coffea arabica]	12.7	0.01194	0.439
NbS00024267g0007.1	Cleft lip and palate transmembrane protein 1 homolog	11.5	0.01424	0.435

NbS00008237g0002.1	Histidinol dehydrogenase	10.1	0.04312	0.434
NbS00010232g0013.1	29 kDa ribonucleoprotein A	36.8	0.03402	0.424
NbS00035939g0004.1	Aspartic proteinase oryzasin-1 precursor	32.3	0.00814	0.423
NbS00000740g0117.1	Putative R3H domain-containing protein 4-like	29.3	0.04974	0.421
NbS00025206g0014.1	Fe-S cluster assembly factor HCF101	24.9	0.02160	0.420
NbS00012577g0009.1	5-methyltetrahydropteroyltriglutamate	49.9	0.00548	0.419
NbS00054224g0001.1	Putative acetyl co-enzyme A carboxylase carboxyltransferase alpha	31.5	0.01596	0.414
NbS00011338g0016.1	GDSL-lipase 1	23.5	0.01380	0.410
NbS00051253g0012.1	L-ascorbate oxidase homolog	21.9	0.02748	0.410
NbS00014176g0002.1	SBT2 protein precursor	18.5	0.00252	0.404
NbS00002350g0011.1	Acetolactate synthase, putative	20.6	0.01652	0.400
NbS00028734g0015.1	20 kDa chaperonin	28.9	0.00858	0.400
NbS00028082g0012.1	Cell division protein FtsZ homolog 1	32.9	0.02782	0.399
NbS00000066g0001.1	Glutaredoxin domain-containing protein	15.5	0.00256	0.397
NbS00025596g0028.1	DNA binding protein, putative	22.0	0.02440	0.396
NbS00015088g0014.1	Uncharacterized GPI-anchored protein	23.0	0.03510	0.391
NbS00054171g0002.1	Protein SSUH2 homolog isoform X2	18.6	0.02262	0.366
NbS00019202g0010.1	Phi-1	16.0	0.03576	0.362
NbS00029129g0005.1	Subtilisin-like protease	11.6	0.02488	0.350
NbS00012311g0014.1	Pyruvate kinase isozyme G	27.6	0.01404	0.337
NbS00033024g0001.1	Multicopper oxidase	6.9	0.00936	0.314
NbS00002255g0005.1	Senescence domain-containing protein	35.0	0.03038	0.300
NbS00023162g0005.1	Non-cell-autonomous protein pathway 2	7.7	0.04484	0.292
NbS00025771g0014.1	Probable polygalacturonase	21.2	0.00850	0.292
NbS00032670g0016.1	Cysteine protease	23.5	0.00660	0.275
NbS00055576g0008.1	PsbP domain-containing protein 5	19.9	0.00648	0.263
NbS00005913g0001.1	Aspartic proteinase isoform 2	23.2	0.00312	0.238
NbS00058562g0005.1	DEAD-box ATP-dependent RNA helicase 3	20.7	0.00796	0.238
NbS00002190g0213.1	Aspartic proteinase	12.6	0.00438	0.225
NbS00032594g0011.1	Dynamin-like protein ARC5	23.0	0.00038	0.217
NbS00025772g0014.1	101 kDa heat shock protein	20.1	0.03572	0.211
NbS00056252g0003.1	Probable xyloglucan endotransglucosylase/hydrolase protein	42.7	0.00008	0.208

[a] Phytozome or NCBI gene accession number

[b] Phytozome or NCBI gene description

[c] The sequences coverage of targeted proteins

[d] The fold change of DAPs compared to PVX-GFP group
