# Supplementary material for: Sugarcane Streak Mosaic Virus P1 Attenuates Plant Antiviral Immunity and Enhances Potato Virus X Infection in Nicotiana benthamiana
Source: Cells. 2022 Sep 14;11(18):2870. doi: 10.3390/cells11182870 (PMC9497147; doi:10.3390/cells11182870)
Supplement: Supplementary file 1 [file cells-11-02870-s001.zip › cells-1854153-supplementary materials/Table S5.pdf]

Supplementary Table S5 List of KEGG pathways of P1^{SCSMV}-induced differently abundant proteins in P1^{SCSMV} heterogeneous expressed *Nicotiana benthamiana* leaf by PVX

KEGG pathways ID[a]	Term description[b]	Protein Accession[c]	Fold[d]	P value	Protein Description[e]
nta00071	Fatty acid degradation	NbS00018357g0005.1	1.570	0.0079	Photosystem I reaction center subunit II, chloroplastic
		NbS00007111g0015.1	1.698	0.0114	Multifunctional protein
		NbS00011268g0007.1	1.748	0.0007	Cytosolic acetoacetyl-coenzyme A thiolase
		NbS00023446g0007.1	1.536	0.0032	3-ketoacyl-CoA thiolase 2, peroxisomal
		NbS00019858g0012.1	2.352	0.0052	Acyl-CoA synthetase
		NbS00043465g0007.1	1.925	0.0021	Long chain acyl-CoA synthetase 8
		NbS00020451g0001.1	1.951	0.0313	3-ketoacyl CoA thiolase 2
		NbS00006179g0007.1	1.715	0.0122	Acyl-coenzyme A oxidase 4, peroxisomal
		NbS00018742g0017.1	2.001	0.0012	3-ketoacyl CoA thiolase 1
nta00940	Phenylpropanoid biosynthesis	NbS00003851g0014.1	11.121	0.0001	Peroxidase
		NbS00010850g0119.1	4.489	0.0003	Chloramphenicol acetyltransferase-like domain
		NbS00002272g0002.1	1.572	0.0107	Cinnamate-4-hydroxylase 1
		NbS00037999g0007.1	2.457	0.0216	Phenylalanine ammonia-lyase 1
		NbS00003479g0020.1	2.101	0.0003	Catechol O-methyltransferase
		NbS00058677g0004.1	0.535	0.0446	Beta-D-glucan exohydrolase
		NbS00019371g0003.1	3.193	0.0180	Peroxidase
		NbS00058225g0005.1	6.282	0.0004	Peroxidase
		NbS00012136g0003.1	0.603	0.0431	Beta-D-glucan exohydrolase
		NbS00018998g0013.1	3.62	0.0406	Bacterial-induced peroxidase precursor
nta04146	Peroxisome	NbS00021119g0007.1	1.927	0.0189	2-hydroxyacyl-CoA lyase
		NbC26053601g0004.1	1.77	0.0284	Superoxide dismutase [Cu-Zn]
		NbS00013764g0007.1	5.494	0.0026	Catalase isozyme 1
		NbS00016456g0007.1	2.934	0.0007	Reductases with broad range of substrate specificities
		NbS00011268g0007.1	1.536	0.0032	3-ketoacyl-CoA thiolase 2, peroxisomal
		NbS00019858g0012.1	1.925	0.0021	Long chain acyl-CoA synthetase 8
		NbS00051044g0009.1	3.017	0.0027	Short-chain dehydrogenase
		NbS00043465g0007.1	1.951	0.0313	3-ketoacyl CoA thiolase 2
		NbS00020451g0001.1	1.715	0.0122	Acyl-coenzyme A oxidase 4, peroxisomal
		NbS00006179g0007.1	2.001	0.0012	3-ketoacyl CoA thiolase 1
nta00592	Alpha-Linolenic acid metabolism	NbS00011268g0007.1	1.536	0.0032	3-ketoacyl-CoA thiolase 2, peroxisomal
		NbS00018357g0005.1	1.698	0.0114	Multifunctional protein
		NbS00020451g0001.1	1.715	0.0122	Acyl-coenzyme A oxidase 4, peroxisomal
		NbS00043465g0007.1	1.951	0.0313	3-ketoacyl CoA thiolase 2
		NbS00018742g0017.1	2.272	0.0064	Hydroxyacyl-CoA dehydrogenase
		NbS00006179g0007.1	2.001	0.0012	3-ketoacyl CoA thiolase 1
nta00196	Photosynthesis - antenna proteins	NbS00004910g0006.1	1.63	0.0373	Chlorophyll a/b-binding protein
		NbS00029431g0007.1	1.786	0.0136	Chlorophyll a-b binding protein 13, chloroplastic
		NbS00002044g0005.1	1.669	0.0478	Chlorophyll a/b binding protein
		NbS00004085g0015.1	1.563	0.0236	Chloroplast pigment-binding protein CP26

		NbS00050671g0012.1	1.691	0.0032	Chlorophyll a-b binding protein 36, chloroplastic
nta01040	Biosynthesis of unsaturated fatty acids	NbS00011268g0007.1	1.536	0.0032	3-ketoacyl-CoA thiolase 2, peroxisomal
		NbS00020451g0001.1	1.715	0.0122	Acyl-coenzyme A oxidase 4, peroxisomal
		NbS00043465g0007.1	1.951	0.0316	3-ketoacyl CoA thiolase 2
		NbS00006179g0007.1	2.001	0.0012	3-ketoacyl CoA thiolase 1
nta04016	MAPK signaling pathway	NbS00061216g0001.1	18.169	0.0044	Pathogenesis-related protein 1
		NbS00023821g0011.1	15.126	0.0020	Endochitinase A
		NbS00000307g0001.1	8.507	0.0266	Defense-related protein containing SCP domain
		NbS00013764g0007.1	5.494	0.0026	Catalase isozyme 1
		NbS00024411g0009.1	4.236	0.0158	Acidic endochitinase Q
		NbS00010078g0005.1	3.089	0.0407	Chitinase 134

[a] The identifier in reference KEGG pathways of *N. benthamiana*

[b] Name of KEGG pathway enriched with the *N. benthamiana* proteins

[c] Accession number in the *N. benthamiana* protein database

[d] The regulated folds of DAPs compared to PVX-GFP group.

[e] The basic function description of enriched protein
