# Supplementary material for: Sugarcane Streak Mosaic Virus P1 Attenuates Plant Antiviral Immunity and Enhances Potato Virus X Infection in Nicotiana benthamiana
Source: Cells. 2022 Sep 14;11(18):2870. doi: 10.3390/cells11182870 (PMC9497147; doi:10.3390/cells11182870)
Supplement: Supplementary file 1 [file cells-11-02870-s001.zip › cells-1854153-supplementary materials/Table S6.pdf]

Supplementary Table S6 -Validation of DAPs from P1[SCSMV] heterologous expressed *N. benthamiana* plants obtained from 4D-lable free proteomic by qRT-PCR and western blotting

N. benthamiana **database Protein ID[a]**	***N. benthamiana*** **database Transcription ID[b]**	**Protein description[c]**	**High/Low abundance**	**Protein Proteomic**		**qRT-PCR**	
				**Fold change**	**P value**	**Fold change**	***p*-value**
NbS00004447g0101.1	Niben101Scf03993g05005.1	5-epi-aristolochene synthase-like (EAS)	High	57.252	0.0012	24.8274	0.0335
NbS00023821g0011.1	Niben101Scf07491g00003.1	Endochitinase A	High	15.126	0.0020	23.9518	0.0254
NbS00061216g0001.1	Niben101Scf04053g02006.1	Pathogenesis-related protein 1 like (PR-1-like)	High	18.169	0.0044	23.587	0.1253
NbS00013764g0007.1	Niben101Scf14996g00009.1	Catalase (CAT)	High	5.494	0.0026	3.018	0.0258
NbS00003017g0007.1	Niben101Scf10254g01002.1	Glutathione S-transferase U9-like (GST)	High	3.754	0.0070	3.112	0.0589
NbS00052235g0008.1	Niben101Scf03026g01009.1	L-ascorbate oxidase-like isoform X2 (ASO)	High	2.824	0.0018	2.864	0.0078
NbS00023329g0001.1	Niben101Scf06266g00002.1	Lycopene beta cyclase, chloroplastic	Low	0.646	0.0301	0.1531	0.0584
NbS00028082g0012.1	Niben101Scf07163g00020.1	Cell division protein FtsZ homolog 1 (FtsZ)	Low	0.399	0.0278	0.2305	0.0694
NbS00055576g0008.1	Niben101Scf00777g04013.1	PsbP domain-containing protein 5 (PsbP-like)	Low	0.263	0.0065	0.1463	0.0596
NbS00032594g0011.1	Niben101Scf08364g00011.1	Dynamin-like protein ARC5 isoform (ARC5)	Low	0.217	0.0004	0.0186	0.0235
NbS00002190g0213.1	Niben101Scf04084g00005.1	Aspartic proteinase (ASP)	Low	0.225	0.0044	0.0073	0.0002
NbS00056252g0003.1	Niben101Scf00369g05011.1	Xyloglucan endotransglucosylase (XTH1)	Low	0.208	0.0009	0.0010	0.0025

Note: * represents most significant DAPs

[a] The accession number of *N. benthamiana* protein database.

[b] The accession number of *N. benthamiana* transcripts database.

[c] Target gene description.
